# Supplementary material for: Saccharomyces boulardii (CNCM I-745) improves intestinal damage in sepsis by remodeling intestinal flora structure
Source: Front Cell Infect Microbiol. 2025 Jul 31;15:1602792. doi: 10.3389/fcimb.2025.1602792 (PMC12350329; doi:10.3389/fcimb.2025.1602792)
Supplement: Supplementary file 1 [file DataSheet1.zip › summary/summary/7_Differential_analysis/2_LEfSe_result/Group_BW4_vs_BW0_0.05_3.0/LEfSe_Group_BW4_vs_BW0.abund.pdf]

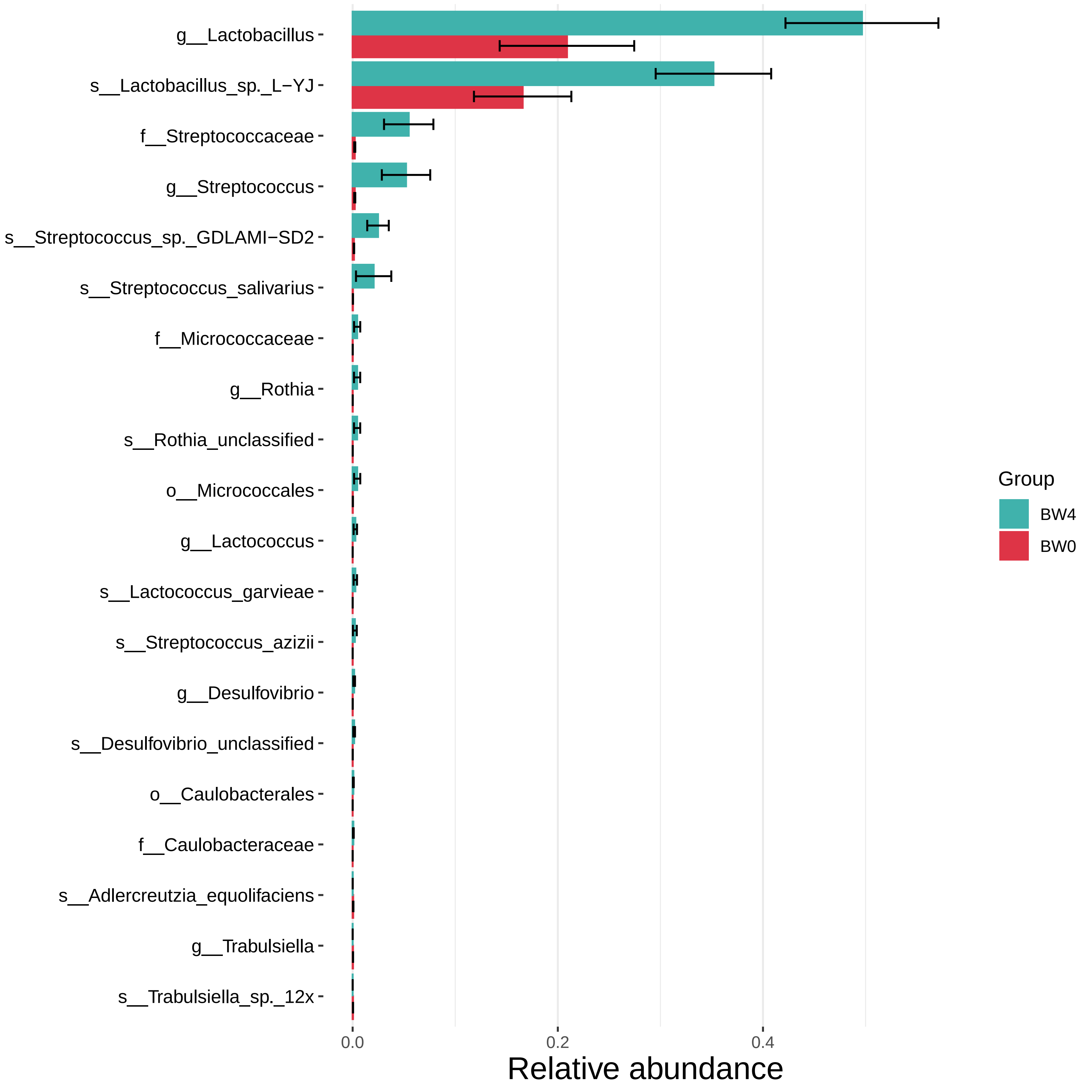

g__Lactobacillus
s__Lactobacillus_sp._L−YJ
f__Streptococcaceae
g__Streptococcus
s__Streptococcus_sp._GDLAMI−SD2
s__Streptococcus_salivarius
f__Micrococcaceae
g__Rothia
s__Rothia_unclassified
o__Micrococcales
g__Lactococcus
s__Lactococcus_garvieae
s__Streptococcus_azizii
g__Desulfovibrio
s__Desulfovibrio_unclassified
o__Caulobacterales
f__Caulobacteraceae
s__Adlercreutzia_equolifaciens
g__Trabulsiella
s__Trabulsiella_sp._12x
0.0
0.2
0.4
Relative abundance
Group
BW4
BW0
